# Calcium and phosphorus supplemented diet increases bone volume after thirty days of high speed treadmill exercise in adult mice

**DOI:** 10.1038/s41598-022-19016-8

**Published:** 2022-08-26

**Authors:** Michael A. Friedman, David H. Kohn

**Affiliations:** The University of Michigan, 1011 N University Ave., Ann Arbor, MI 48109 USA

**Keywords:** Biomedical engineering, Preclinical research, Bone

## Abstract

Weight-bearing exercise increases bone mass and strength. Increasing bone loading frequency during exercise can strengthen bone. Combining exercise with a calcium- and phosphorus-supplemented diet increases cortical area more than exercise alone in mice. Thus, we hypothesized that combining high-speed treadmill exercise while feeding mice a mineral-supplemented diet would lead to greater cortical area than high-speed exercise on a standard diet and low-speed exercise on a supplemented diet. Fifteen-week old male C57BL/6 mice were assigned to seven groups—(1) baseline, (2) non-exercise fed a control diet, (3) non-exercise fed a supplemented diet, (4) low-speed exercise fed a control diet, (5) low-speed exercise fed a supplemented diet, (6) high-speed exercise fed a control diet, and (7) high-speed exercise fed a supplemented diet. Mice exercised thirty days for 20 min/day at 12 m/min or 20 m/min. Tibiae were assessed by micro-CT and 4-point bending. Cortical area fraction and trabecular bone volume fraction (BV/TV) were significantly increased by the supplemented diet. High-speed exercised mice had significantly lower body weight, with no detrimental effects to bone health. Increasing running speed can decrease body weight while maintaining the benefits of exercise and nutrition on bone health. Running can lower body weight without harming bone health.

## Introduction

Weight-bearing exercise in humans and mice can increase bone mass, structural (whole bone)-level strength, and/or tissue quality, making bone better able to resist fracture^1–4^. Exercise can also have long-term benefits to bone health even after detraining^5–13^. In humans, bone mass peaks at ~ 25 years of age and declines thereafter. It is therefore beneficial to maximize bone mass accumulation early in life to maintain a higher level of bone mass in old age when weight-bearing exercise becomes more difficult to perform^14–16^.

Bone responds to loading from exercise by increasing bone mass and/or bone strength to accommodate greater loads and prevent damage^17^. Exercise in humans and rodents can increase bone mass by increasing the magnitude of strain on the bone, leading to increases in bone cross-sectional area^18^ and bone formation rate^19,20^. Loads generating greater strain magnitudes increase bone formation rate when applied at a higher loading frequency^21^. Higher-intensity exercise regimens achieved by increasing treadmill speed may therefore be able to increase bone formation.

Increasing bone formation may allow bone to reach greater peak bone mass or to achieve peak bone mass in a shorter time, allowing for a more rapid increase in resistance to fracture. Maximizing bone mass with exercise could require an increased dietary mineral supply to simultaneously increase bone mass and bone tissue quality. Since exercise increases demand for dietary minerals^22^, treadmill exercise may cause an even greater need for minerals that normal dietary amounts cannot provide. Combining higher-speed treadmill exercise with a calcium- and phosphorus-supplemented diet may further increase bone mass beyond what standard, lower-speed exercise can achieve.

Under standard exercise conditions (running at a speed similar to jogging), rodents exercised for 6–12 weeks have increased cortical bone mineral content, area, yield force, and ultimate force^13,22–25^. In young adult mice, combining exercise with a mineral-supplemented diet increases tibial cortical area compared to exercise with a standard diet after only 3 weeks^2^. It was therefore hypothesized that combining a mineral-supplemented diet with high-speed treadmill exercise would lead to greater cortical area than high-speed exercise on a standard diet after a relatively short exercise program of thirty days in young adult mice.

## Results

### High-speed exercise prevented weight gain

**Figure 1:**
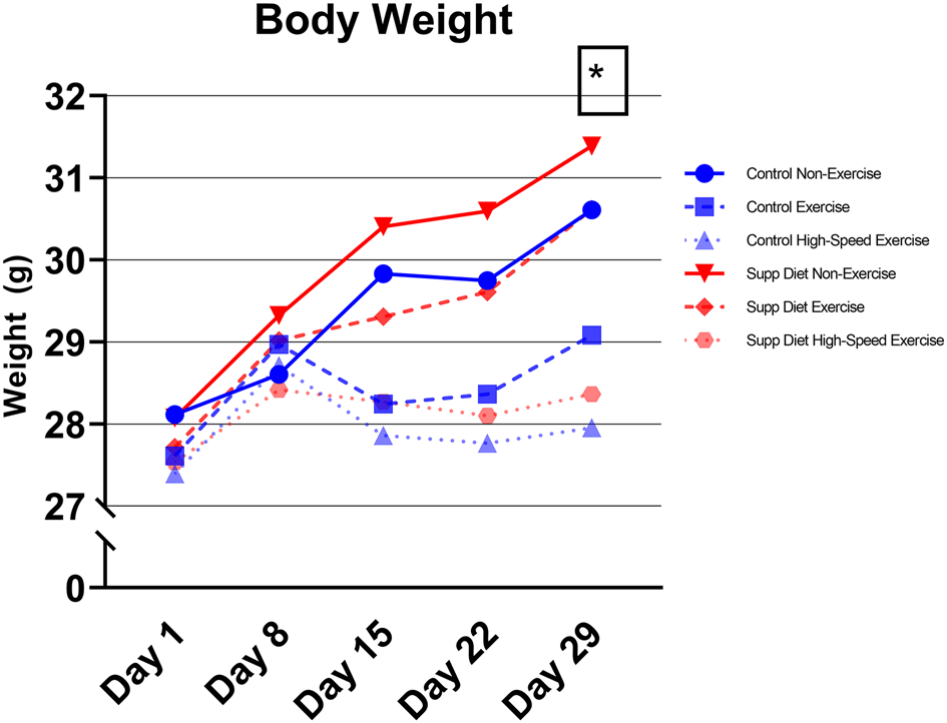
Mouse body weight (mean ± SD, n = 20/group). High-speed exercise limited gains in body weight after one week. On day 29, both of the high-speed exercised groups and the low-speed exercised group on the control diet had lower body weight than the non-exercised groups and the exercised group on the supplemented diet. *Significant exercise effect on Day 29 (*p* < 0.05, Two-way ANOVA).

There was a significant main effect of exercise on body weight after thirty days (*p* < 0.05, Two-way ANOVA, Fig. 1). Mice in both of the high-speed exercise groups did not gain weight after day 8. The same effect on body weight occurred in mice exercised at low-speed while fed the control diet. Body weight in all other groups of mice increased continuously after day 8. Mice in these groups finished the study with significantly higher body weight than the mice in the three groups that did not gain weight after day 8 (*p* < 0.05, Tukey’s tests).

### Mineral supplemented diet increased tibial cortical area after thirty days

**Figure 2:**
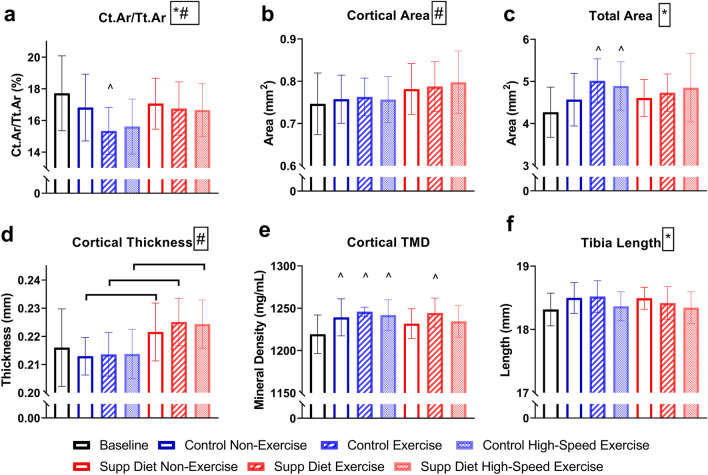
Mouse left tibial mid-diaphyseal cortical bone cross-sectional geometric properties and mineralization (mean ± SD, n = 20/group). Diet significantly increased cortical area fraction, cortical area, and thickness. Exercise significantly decreased cortical area fraction and tibial length. ^#^Significant diet effect (*p* < 0.05, Two-way ANOVA). *Significant exercise effect (*p* < 0.05, Two-way ANOVA). ^Significantly different from baseline (*p* < 0.05, Tukey’s-test). Horizontal bar represents significant difference between groups (*p* < 0.05, Tukey’s test).

Diet had a significant main effect on cortical area fraction (Ct.Ar/Tt.Ar), cortical area (Ct.Ar), and cortical thickness (Ct.Th) after thirty days (*p* < 0.05, Two-way ANOVA, Fig. 2). Exercise had a significant main effect on Ct.Ar/Tt.Ar, total cross-sectional area, and tibial length (*p* < 0.05, Two-way ANOVA). All mice on the supplemented diet had significantly greater cortical thickness than mice on the control diet (*p* < 0.05, Tukey’s tests). Low-speed exercised mice on the control diet had significantly lower Ct.Ar/Tt.Ar than baseline mice (*p* < 0.05, Tukey’s tests). Tissue mineral density was significantly greater than baseline in all groups on the control diet and in low-speed exercised mice on the supplemented diet (*p* < 0.05, Tukey’s tests). There were no significant differences between high-speed and low-speed exercise for cortical bone properties.

### The supplemented diet increased trabecular bone volume fraction from baseline

**Figure 3:**
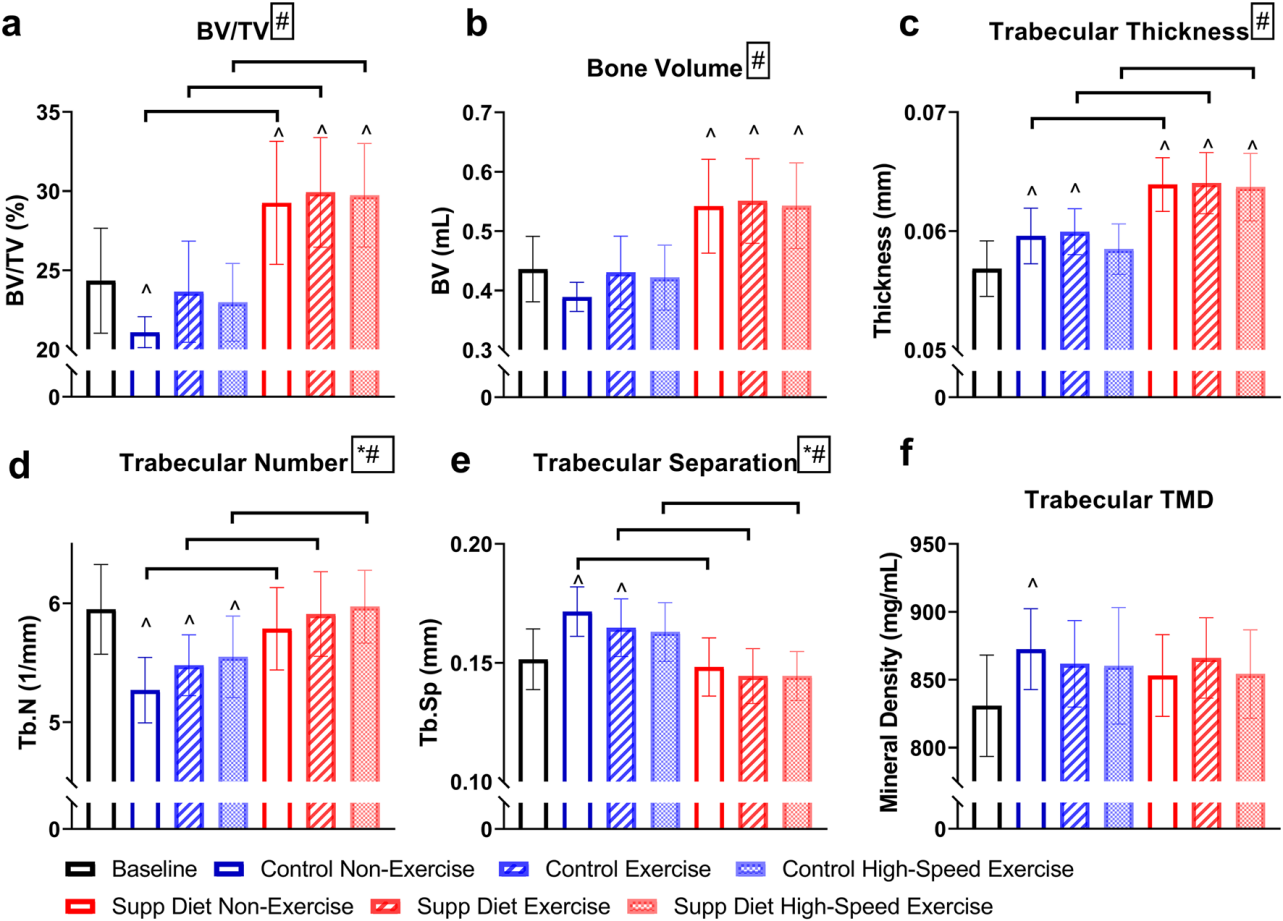
Proximal left tibial trabecular architecture (mean ± SD, n = 20/group). Diet had a significant main effect on every measured property except tissue mineral density (TMD), increasing trabecular bone volume (BV), bone volume/total volume (BV/TV), trabecular number (Tb.N), and trabecular thickness (Tb.Th) and decreasing trabecular separation (Tb.Sp). For mice fed the control diet, both speeds of exercise prevented a significant decrease in BV/TV from baseline. #Significant diet effect (*p* < 0.05, Two-way ANOVA). *Significant exercise effect (*p* < 0.05, Two-way ANOVA). ^Significantly different from baseline (*p* < 0.05, Tukey’s test). Horizontal bar represents significant difference between groups (*p* < 0.05, Tukey’s test).

Diet had a significant main effect on trabecular BV/TV, BV, Tb.Th, Tb.N, and Tb.Sp (*p* < 0.05, Two-way ANOVA, Fig. 3). Exercise had a significant main effect on Tb.N and Tb.Sp. Each group on the supplemented diet had significantly greater trabecular BV/TV, Tb.N, and Tb.Th and significantly lower Tb.Sp than the control diet group subjected to the same exercise speed (*p* < 0.05, Tukey’s tests). All groups on the supplemented diet had significantly greater BV/TV, BV, and Tb.Th than baseline (*p* < 0.05, Tukey’s tests). The non-exercised mice on the control diet had significantly lower BV/TV than baseline (*p* < 0.05, Tukey’s tests). Both low- and high-speed exercise prevented this decline.

### The supplemented diet increased yield force, and exercise decreased ultimate stress

**Figure 4:**
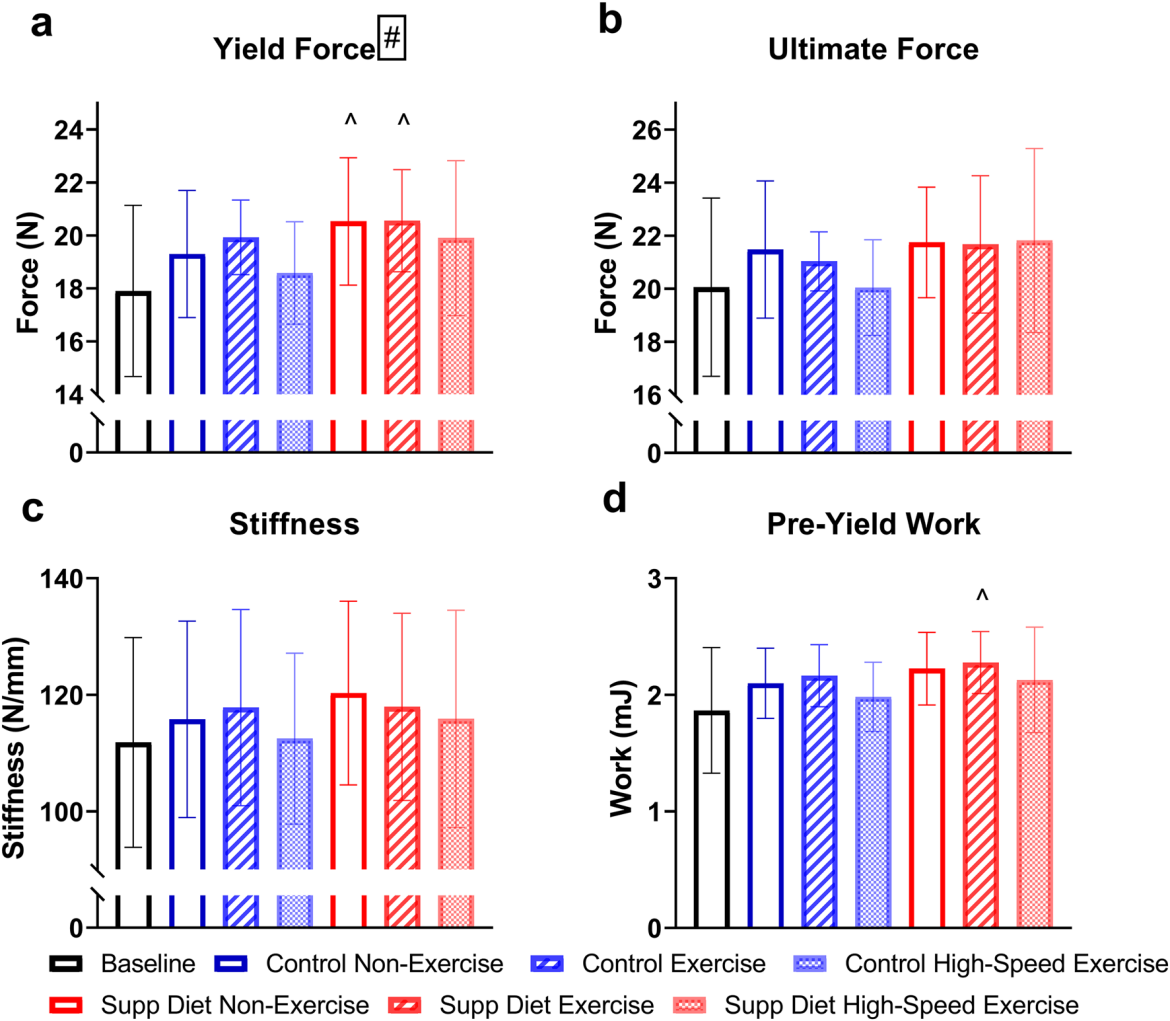
Structural-level left tibial mechanical properties (mean ± SD, n = 20/group). There was a significant main effect of diet on yield force, and non-exercised mice on the supplemented diet and mice exercised at low speed on the supplemented diet had significantly greater yield force than baseline. #Significant diet effect (*p* < 0.05, Two-way ANOVA). ^Significantly different from baseline (*p* < 0.05, Tukey’s test).

**Figure 5:**
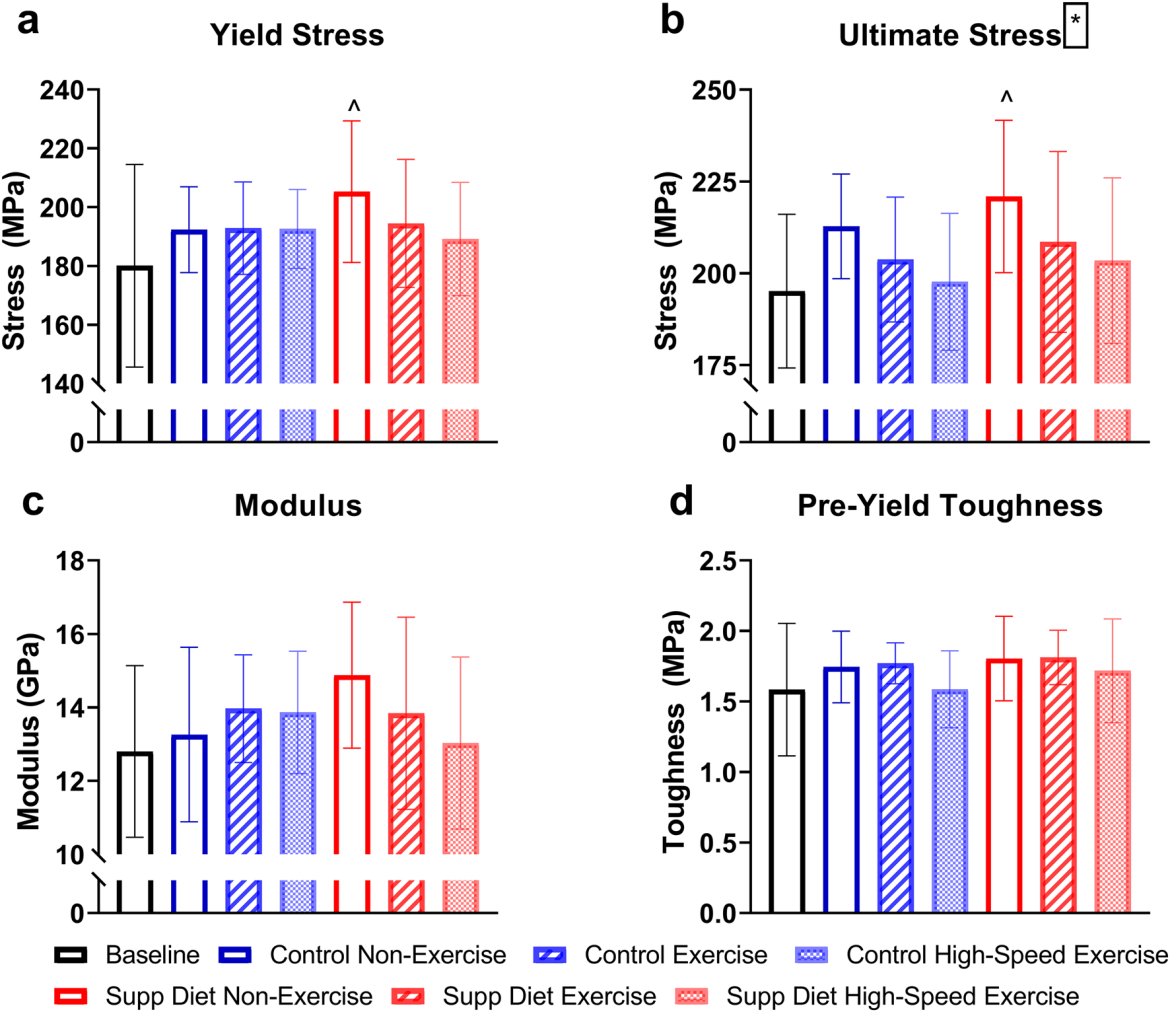
Tissue-level left tibial mechanical properties (mean ± SD, n = 20/group). Exercise had a significant main effect on ultimate stress, decreasing bone strength. Yield stress and ultimate stress were significantly greater than baseline in non-exercised mice fed the supplemented diet. *Significant exercise effect (*p* < 0.05, Two-way ANOVA). ^Significantly different from baseline (*p* < 0.05, Tukey’stest).

At the structural (whole bone)-level, diet had a significant main effect on yield force after thirty days (*p* < 0.05, Two-way ANOVA, Fig. 4). Low-speed exercised mice on the supplemented diet had significantly greater yield force and pre-yield work than baseline (*p* < 0.05, Tukey’s tests), and non-exercised mice on the supplemented diet had significantly greater yield force than baseline. There were no significant main effects of exercise or significant group differences for any structural-level mechanical property. At the tissue-level, exercise had a significant main effect on ultimate stress after thirty days (*p* < 0.05, Two-way ANOVA, Fig. 5). The non-exercised mice on the supplemented diet had significantly greater yield stress and ultimate stress than baseline (*p* < 0.05, Tukey’s tests). There were no significant main effects of diet on tissue-level mechanical properties.

### The supplemented diet increased serum Ca throughout the study while exercise only increased serum Ca on day 30

**Figure 6:**
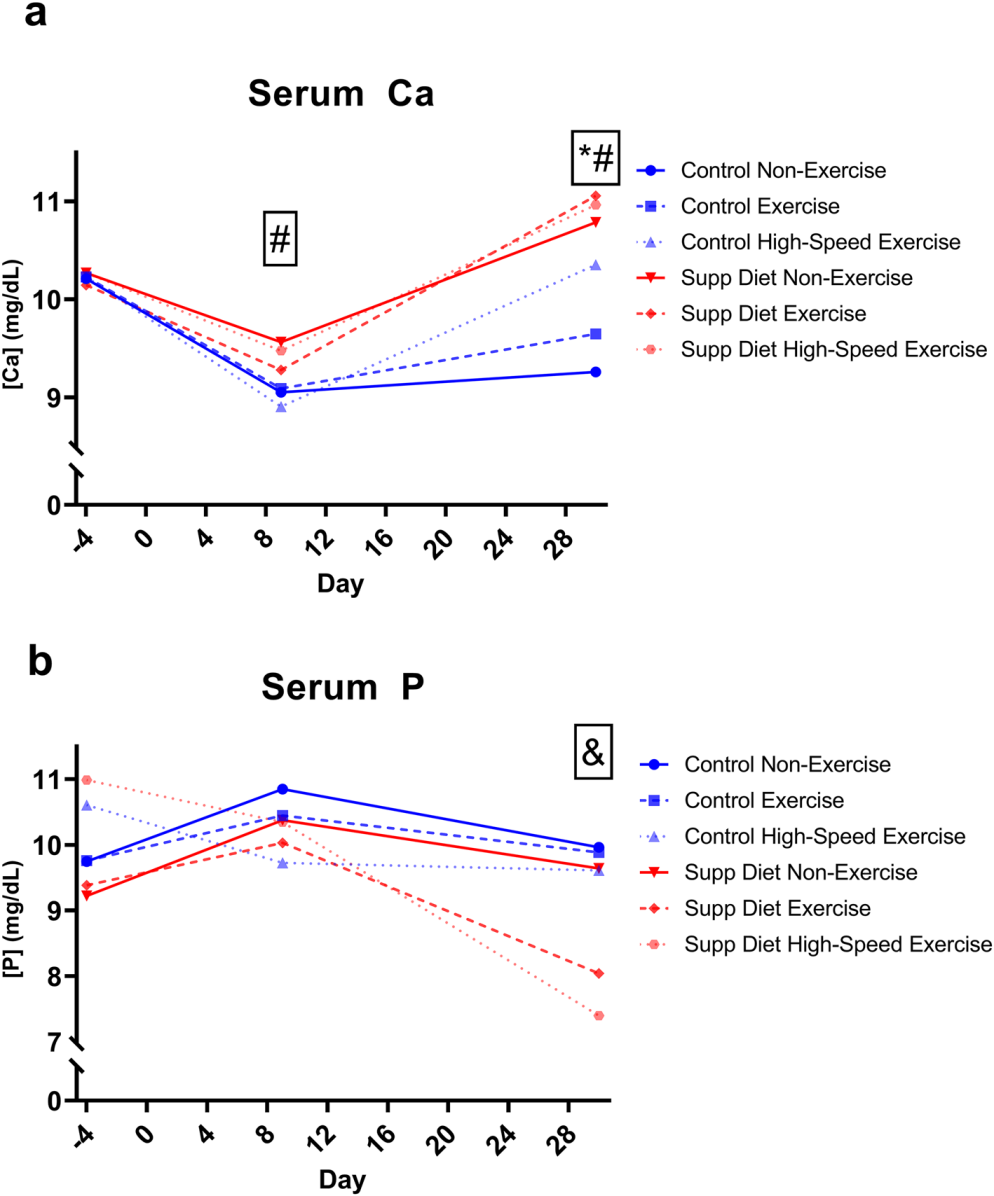
Mean serum [Ca] and [P] on days -4, 9, and 30, n = 20/group. Error bars removed for clarity. The supplemented diet significantly increased serum Ca on days 9 and 30. Exercise only significantly increased serum Ca on day 30. Diet and exercise had a significant interactive effect on serum P on day 30. #Significant diet effect on that day (*p* < 0.05, Two-way ANOVA). *Significant exercise effect on that day (*p* < 0.05, Two-way ANOVA). &Significant diet and exercise interaction on that day (*p* < 0.05, Two-way ANOVA).

There was a significant main effect of diet on day 9 and day 30 serum Ca (*p* < 0.05, Two-way ANOVA, Fig. 6). There was a significant main effect of exercise on serum Ca only on day 30. For mice on the control diet, high-speed exercise significantly increased serum Ca on day 30 (*p* < 0.05, Tukey’s test). For mice on the supplemented diet, there were no significant effects of exercise on serum Ca. There were no significant effects of diet or exercise on serum P on day 9. On day 30, there was a significant diet and exercise interaction on serum P. Both high- and low-speed exercised groups on the supplemented diet had significantly lower day 30 serum P than all other groups.

### Exercise decreased PINP/CTX ratio in mice on the control diet on day 9 and mice on the supplemented diet on day 30

**Figure 7:**
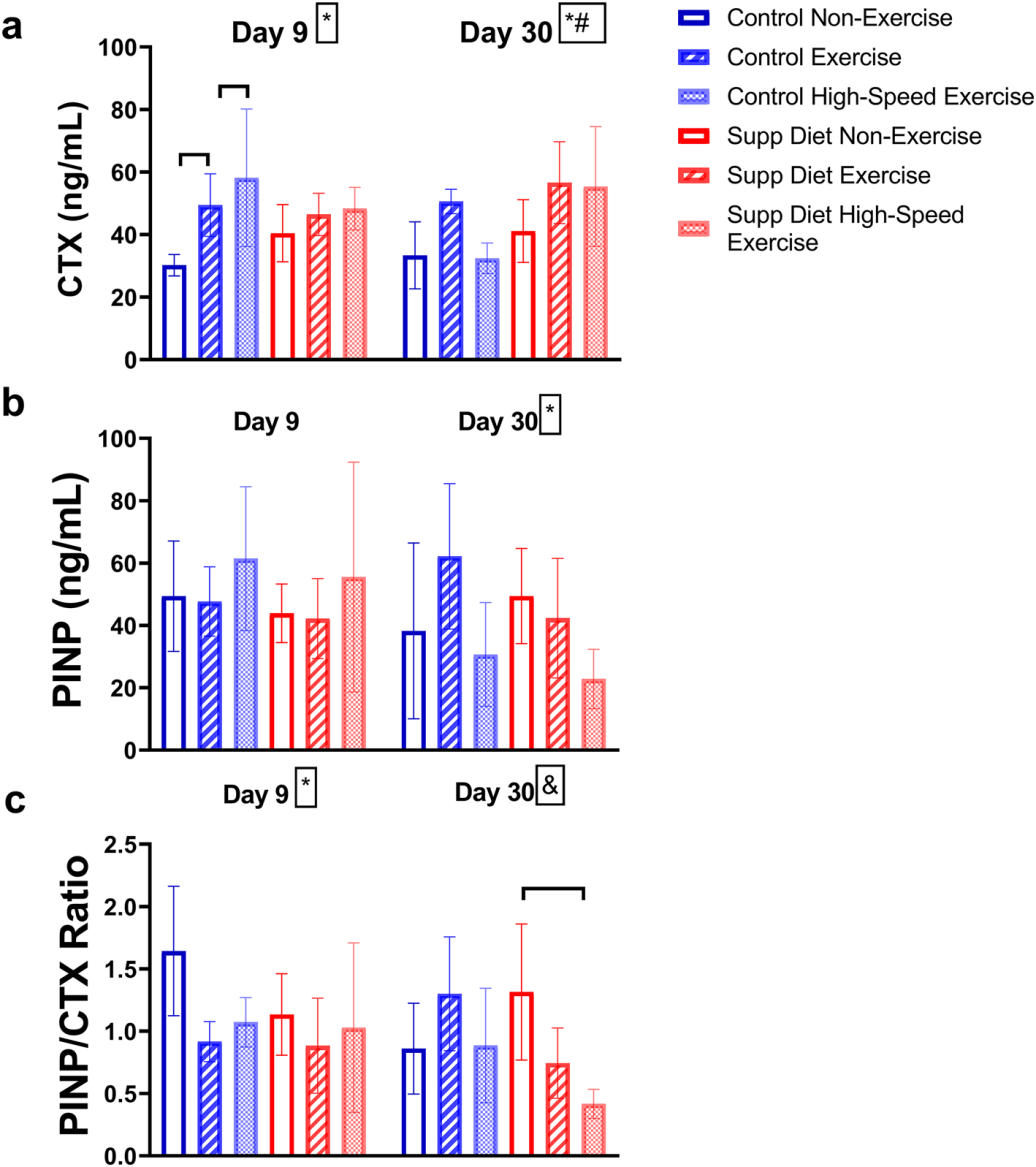
Serum CTX and PINP (mean ± SD, n = 7/group) on day 9 and day 30. For mice on the control diet, exercise significantly decreased PINP/CTX on day 9, causing a less formation-favored state of bone metabolism. Similarly, for mice on the supplemented diet, exercise significantly decreased PINP/CTX on day 30. #Significant diet effect (*p* < 0.05, Two-way ANOVA). *Significant exercise effect (*p* < 0.05, Two-way ANOVA). &Significant diet and exercise interaction on that day (*p* < 0.05, Two-way ANOVA). Horizontal bar represents significant difference between groups (*p* < 0.05, Tukey’s test).

Exercise had significant main effects on serum CTX and the PINP/CTX ratio on day 9 (*p* < 0.05, Two-way ANOVA, Fig. 7). Mice on the control diet subjected to both low- and high-speed exercise had significantly higher day 9 CTX than non-exercised mice on the control diet (*p* < 0.05, Tukey’s test). There were no significant main effects of diet, suggesting exercise was more impactful on bone metabolism than diet on day 9. There were significant main effects of exercise on serum CTX and PINP on day 30, and there was a significant main effect of diet on serum CTX on day 30. On day 30, high-speed exercised mice on the supplemented diet had significantly lower serum PINP/CTX ratio than non-exercised mice on the supplemented diet.

## Discussion

After thirty days of interventions, diet had a greater impact on bone mass and strength than exercise (Figs. 2, 3 and 4). The diet and exercise regimen in this study (4 weeks, 20 min exercise per day) resulted in similar effects on tibial bone mass and strength as previous studies that involved longer durations of exercise (8 weeks, 30 min exercise per day)^2,5^. Direct tibial loading in rodents has diminished effectiveness when applied for a large number of load cycles per day, but the number of loading cycles used here was an order of magnitude greater^26^. Thus, longer durations of treadmill exercise are not likely to be more beneficial to bone health.

The diet and exercise interventions were most impactful on tibial trabecular bone. This study showed, for the first time, that this supplemented diet increases trabecular bone volume in the proximal tibia (Fig. 3B). The increased BV/TV, but not CtAr/TtAr, cortical area, or TMD in mice on the supplemented diet may be a sign that this short-term diet and exercise program was more effective on trabecular bone. The response of bone to mechanical and other stimuli can be site specific^27^. Changes in trabecular, but not cortical bone after a short intervention could occur because trabecular bone metabolic rate can be higher than in cortical bone. Longer-term exercise for 8 weeks increases cortical area^2^, and the high-speed exercise program used here may also have increased cortical area if the exercise program duration was increased.

There was a decline in trabecular BV/TV with age from 15 to 19 weeks old in control mice. For mice on the control diet, exercise prevented this age-related decline in trabecular BV/TV. Since exercise appeared to have no effect on mice on the supplemented diet while providing some benefits to mice on the control diet, the effects of exercise may be dependent on dietary mineral supply. Exercise appears to be most impactful when dietary mineral supply is insufficient.

High-speed exercise significantly decreased body weight by the end of the study (Fig. 1). Mice in the high-speed exercise groups did not have lower tibial bone mass or mechanical strength, as might be expected with decreased body weight^28,29^. Normalizing bone mass and strength by body weight did not reveal any additional insights, as no body-weight normalized properties were significantly affected by exercise (data not shown). Thus, the high-speed exercise program allowed mice to reach the same bone mass and strength at a lower body weight compared to non-exercised mice.

The supplemented diet caused significantly greater serum Ca than the control diet on day 9 and day 30 (Fig. 6A). The greater serum supply of Ca from the supplemented diet ultimately could have led to greater trabecular BV/TV after thirty days. Since exercise only affected serum Ca at the end of the study, increasing the duration of the exercise program could lead to further increases in trabecular BV/TV in exercised mice on the standard diet.

Mice exercised at both high and low speeds while fed the supplemented diet had significantly lower day 30 serum phosphorus than non-exercised mice and mice exercised while fed the control diet (Fig. 6B). Rodents subjected to treadmill exercise can have increased demand for phosphorus as well as calcium^30^. Although the supplemented diet has twice the phosphorus as the control diet, this concentration may still not be sufficient for the increased mineral demands from exercise. Exercise did not decrease serum phosphorus for mice on the control diet, possibly due to the lower Ca:P ratio in the control diet. The elevated Ca:P ratio in the supplemented diet may be beneficial towards serum calcium, but detrimental towards serum phosphorus. Mice were not assigned to groups of equivalent baseline serum phosphorus. Initial differences in serum phosphorous may have been a factor in the significant group differences seen on day 30, though only the exercised mice on the supplemented diet had a decrease in serum phosphorus from day 9 to day 30.

On day 9, there was a significant main effect of exercise on the PINP/CTX ratio (Fig. 7C). This change in bone metabolism would be expected to lead to decreased bone formation. Similar to the reduction in PINP after one day of exercise^2^, lower PINP/CTX on day 9 suggests exercise could be limiting bone growth for more than a week after the start of the exercise program. This decrease in growth is transient, as there are no significant differences in cortical area and trabecular bone volume in exercised mice after thirty days (Figs. 2B and 3B). For mice on the supplemented diet, on day 30, high-speed exercise significantly decreased the PINP/CTX ratio (Fig. 7C). Mice exercised while on the supplemented diet may be reaching peak bone mass at a faster rate than non-exercised mice on the same diet. This would lead to a decline in PINP/CTX ratio as was seen in this study.

There was no significant effect of exercise speed on any morphological or mechanical measurements of either cortical or trabecular bone. Increasing treadmill speed may not be impactful on bone if it does not lead to greater peak loads. High-speed exercise did lead to greater average loading frequency, but may not have been a great enough change to have an effect. In the direct tibial loading model, increasing loading frequency is associated with increased bone formation rate^21^. Bone formation rate was not directly measured here, but the bone metabolism markers CTX, PINP, and PINP/CTX can undergo changes that are indicative of increased bone formation^2,31,32^.

There were some limitations to this study. We were unable to measure the magnitude of peak strain applied to the bones during exercise. Thus, we could not determine whether increasing treadmill speed led to increased strain magnitude on the bones. However, we measured increased mouse step rate at the higher speed of exercise, indicating an increase in strain rate was likely being applied to the bones. In order to compare to previous work, only adult male C57BL/6 mice were used. Thus, we don’t know whether mouse age, sex, and/or strain impact the results of these diet and exercise interventions. Future work could examine the effects of these interventions in different populations of mice at different durations of diet and exercise. Additionally, food consumption and mouse cage activity levels were not measured, so it is not known whether the loss of body weight is due to the increase in activity or decrease in food consumption. Mice running at speeds similar to our high-speed treadmill exercise can have decreased food consumption^33,34^. It’s possible that high-speed exercised mice had decreased food consumption, resulting in decreased body weight. However, all bone mass and strength measurements indicated bone mass and strength were increased and maintained, respectively. Thus, it appears that all high-speed exercised mice received sufficient nutrient intake for bone health. This study was done in mice that run using four legs. The results may vary in humans which run on two legs and may have different distribution of loading on the bones during running.

After thirty days of high-speed treadmill exercise, mice had lower body weight, regardless of dietary mineral supply. These changes in weight did not come at the expense of tibial cortical bone mass, trabecular bone volume, or mechanical properties. High-speed exercised mice on the supplemented diet had the greatest CtAr/TtAr, cortical area, and trabecular BV/TV after thirty days. Together, these changes in bone properties and body weight suggest combining a high-speed exercise program with a mineral-supplemented diet could be best for maximizing bone mass and strength to prevent bone fractures in humans, along with additional health benefits of weight loss. These results could lead to the design of diet and exercise interventions for weight loss without detrimental effects to bone health.

## Methods

### Animals and treatments

All animal procedures were done with the approval of the University of Michigan University Committee on Use and Care of Animals (protocol #00005237) and complied with the relevant guidelines and regulations. This study is reported in accordance with ARRIVE guidelines. One hundred thirty-eight male C57BL/6 mice, 26.3 ± 2.8 g mean body weight, were purchased from Charles River Laboratories (Wilmington, MA) at 13 weeks of age. The mice were single housed, and they were fed the control diet for 2 weeks of acclimation. On day 1, at 15 weeks of age, mice were assigned to one of 7 groups—(1) a baseline group sacrificed on day 1, (2) a non-exercise group fed the control diet, (3) a non-exercise group fed the supplemented diet, (4) a low-speed exercise group fed the control diet, (5) a low-speed exercise group fed the supplemented diet, (6) a high-speed exercise group fed the control diet, and (7) a high-speed exercise group fed the supplemented diet. Mice were divided into groups of equal mean body weight and baseline serum Ca concentration. Baseline serum Ca was measured 5 days before experiment day 1 (day -4). After 30 days of treatment(s), mice from all of the experimental groups were sacrificed at 19 weeks of age, and left tibiae were harvested for analysis.

### Diets and exercise program

Diet formulations were the same as we have used previously^2,5^. The control diet was an AIN-93G diet (TestDiet®, Richmond, IN) that was modified by adding dicalcium phosphate to contain 0.5% Ca and 0.5% P. In the supplemented diet, dicalcium phosphate and calcium carbonate were added to give the diet 5% Ca and 1% P. Dietary mineral amounts and Ca:P ratio were all designed to increase serum Ca by increasing passive absorption of Ca in the intestines^2,5,35–37^. For the control diet, the food had 3.90 kcal/g, and the calorie distribution was 65.0% carbohydrates, 16.3% fat, and 18.7% protein, while the supplemented diet had 3.39 kcal/g with the same calorie distribution. All other nutrients were equivalent between the diets.

All exercise was performed by treadmill running during light hours (Columbus Instruments, Model 1055 M, Columbus, OH). The treadmill lanes were covered such that the moving lanes were in the dark, and if mice fell off the treadmill they would be in the light. This provided motivation to stay on the treadmill. The low-speed exercise program consisted of running on a 5° incline treadmill at 12 m/min, 20 min/day for 30 consecutive days. Mice were gradually increased to a maximum speed of 12 m/min in the first 3 days of exercise. The high-speed exercise program consisted of running on a 5° incline treadmill at 20 m/min, 20 min/day. Mice were gradually increased to a maximum speed of 20 m/min in the first 8 days of exercise. This speed was the maximum speed mice could sustain running for 20 min without falling off the treadmill at this incline and is similar to the maximum speed mice can run at^38^. Exercise compliance was maintained by tapping the tails of mice that fell off the treadmill during exercise. Frame-by-frame video analysis of mice running gave an estimated average frequency of 3.4 steps/second in mice running 12 m/min and 4.2 steps/second in mice running 20 m/min. Based on these estimates, mice took an average of 4,080 and 5,040 steps per exercise session in the 12 m/min and 20 m/min speeds, respectively. Thus, the high-speed exercise program had greater loading frequency, and both exercise programs offered a sufficient number of load cycles/day to have an effect on bone such that any further duration would likely not produce additional changes to the bone^26^.

### Cortical geometry and trabecular architecture measurements

Cortical and trabecular bone micro-CT measurements were obtained as we have detailed^5^. Whole tibiae were embedded in agarose, and scanned using a micro-CT specimen scanner (µCT100 Scanco Medical, Bassersdorf, Switzerland). The scan settings were voxel size of 12 μm, 70 kVp, 114 µA, 0.5 mm AL filter, and integration time 500 ms. Scans were analyzed using algorithms in the Scanco IPL software. For measurement of cortical geometry metrics—cortical area fraction (Ct.Ar/Tt.Ar), cortical area (Ct.Ar), total cross-sectional area, cortical thickness (Ct.Th), and volumetric tissue mineral density (TMD), a 180-μm thick transverse section from a standard site located 21.7% of the distance from the tibia-fibula junction to the proximal end of the tibia was chosen. The cortical bone section is located approximately at the center of the four-point bending mechanical testing region. A separate 180-μm thick transverse section at the fracture site was analyzed for cortical geometry measurements used in calculations of tissue-level mechanical properties (moment of inertia, distance from neutral axis). Tibial scans were also analyzed for trabecular bone properties. Proximal tibial metaphyseal sections 480 μm thick from immediately below the growth plate were analyzed using freehand traced volumes of interest. Trabecular properties measured were bone volume fraction (BV/TV), bone volume (BV), trabecular number (Tb.N), trabecular thickness (Tb.Th), trabecular separation (Tb.Sp), and TMD.

### Mechanical testing

Structural- and tissue-level mechanical properties were measured in all groups as we have described previously^2,5^. Structural-level properties (force, deformation, stiffness, work) were measured from a 4-point bending to failure test (3-mm inner and 9-mm outer spans). Tibiae were loaded to failure with the medial side of the mid-diaphysis in tension under displacement control at 0.025 mm/sec at a data sampling rate of 30 Hz. Tissue-level mechanical properties (stress, strain, modulus, toughness) were estimated using beam bending theory with geometric measurements (moment of inertia about anterior–posterior axis, distance from centroid to medial side of the bone) from micro-CT data at the fracture site^39^.

### Serum analysis

Fasting blood samples taken before daily exercise were collected by submandibular vein bleeding as we have described^2^. Mice fasted at the start of light hours, and blood was collected six hours later. Blood samples were collected on day -4 (baseline), day 9 (after the first day of full-speed running for all exercise groups), and on day 30 (the final day of exercise). Our previous work showed significant changes in serum bone metabolism markers occur after one day of exercise, but are not maintained long-term^2^. Thus, serum was examined early in the study, just after high-speed exercised mice began running at full speed for the entire exercise session. Serum was isolated by centrifuge. Calcium and phosphorous concentrations were measured at all time points using the Calcium CPC LiquiColor test kit (Stanbio Laboratory, Boerne, TX) and the Phosphorus Liqui-UV kit (Stanbio Laboratory). ELISAs were used to measure markers of bone formation and resorption—pro-collagen type I amino-terminal peptide (PINP) and carboxy-terminal collagen crosslinks (CTX) (Immunodiagnostic Systems, Inc., Scottsdale, AZ)—on samples from day 9 and day 30. All manufacturers’ kit instructions were followed, including the use of the standards provided for obtaining standard curves.

### Statistical analysis

All statistical analysis was performed using Graphpad Prism. Data was checked for normality using the Shapiro–Wilk test. Cortical geometry measurements, trabecular architecture measurements, mechanical properties, and serum biomarkers were tested by two-way ANOVA with Tukey’s post-hoc tests to determine if the individual effects of diet or exercise were significant (*p* < 0.05) and if the combined treatments had a significant interactive effect. One-way ANOVAs with Tukey’s post-hoc tests were used to compare baseline to experimental groups. Data that failed the normality test was tested using the Kruskal–Wallis test with Dunn’s post-hoc tests. Outliers were removed using the GraphPad ROUT function for detecting outliers using nonlinear regression^40^.

## Supplementary Information

Supplementary Information.

## Data Availability

All data is posted in the manuscript figures and supplemental files.
